# Objective criteria for septal fibrosis in non-ischemic dilated cardiomyopathy: validation for the prediction of future cardiovascular events

**DOI:** 10.1186/s12968-016-0300-z

**Published:** 2016-11-14

**Authors:** Yoko Mikami, Aidan Cornhill, Bobak Heydari, Sebastien X. Joncas, Fahad Almehmadi, Mohammed Zahrani, Mahmoud Bokhari, John Stirrat, Raymond Yee, Naeem Merchant, Carmen P. Lydell, Andrew G. Howarth, James A. White

**Affiliations:** 1 Stephenson Cardiac Imaging Centre, Libin Cardiovascular Institute of Alberta, University of Calgary, #0700, SSB, Foothills Medical Centre, 1403-29th St. NW, Calgary, AB T2N2T9 Canada; 2 Department of Medicine, Western University, London, ON Canada; 3 Robarts Research Institute, University of Western Ontario, London, ON Canada; 4 Department of Cardiac Sciences, Libin Cardiovascular Institute, University of Calgary, Calgary, Canada; 5 Department of Diagnostic Imaging, University of Calgary, Calgary, Canada

**Keywords:** Dilated cardiomyopathy, Fibrosis, Cardiovascular magnetic resonance, Prognosis

## Abstract

**Background:**

Expert subjective reporting of mid-wall septal fibrosis on late gadolinium enhancement (LGE) images has been shown to predict major cardiovascular outcomes in patients with non-ischemic dilated cardiomyopathy (NIDCM). This study aims to establish objective criteria for non-experts to report clinically relevant septal fibrosis and compare its performance by such readers versus experts for the prediction of cardiovascular events.

**Methods:**

LGE cardiovascular magnetic resonance (CMR) was performed in 118 consecutive patients with NIDCM (mean age 57 ± 14, 42 % female) and the presence of septal fibrosis scored by expert readers. CMR-naive readers performed signal threshold-based LGE quantification by referencing mean values of remote tissue and applying these to a pre-defined anatomic region to measure septal fibrosis. All patients were followed for the primary composite outcome of cardiac mortality or appropriate implantable cardioverter-defibrillator (ICD) therapy.

**Results:**

The mean LVEF was 32 ± 12 %. At a median follow-up of 1.9 years, 20 patients (17 %) experienced a primary composite outcome. Expert visual scoring identified 55 patients with septal fibrosis. Non-expert septal fibrosis quantification was highly reproducible and identified mean septal fibrosis burden for three measured thresholds as follows; 5SD: 2.9 ± 3.6 %, 3SD: 6.9 ± 6.3 %, and 2SD: 11.1 ± 7.5 % of the left ventricular (LV) mass, respectively. By ROC analysis, optimal thresholds for prediction of the primary outcome were; 5SD: 2.74 % (HR 8.7, *p* < 0.001), 3SD: 6.63 % (HR 5.7, *p* = 0.001) and 2SD: 10.15 % (HR 6.1, *p* = 0.001). By comparison, expert visual scoring provided a HR of 5.3 (*p* = 0.001). In adjusted analysis, objective quantification by a novice reader (>5SD threshold) was the strongest independent predictor of the primary outcome (HR 8.7) and provided improved risk reclassification beyond LVEF alone (NRI 0.54, 95 % CI 0.16–0.92, *p* = 0.005).

**Conclusions:**

Novice readers were able to achieve superior risk prediction for future cardiovascular events versus experts using objective criteria for septal fibrosis in patients with NIDCM. Patients with a septal fibrosis burden >2.74 % of the LV mass (>5SD threshold) were at a 9-fold higher risk of cardiac death or appropriate ICD therapy versus those not meeting this criteria. As such, this study validates reproducible criteria applicable to all levels of expertise to identify NIDCM patients at high risk of future cardiovascular events.

## Background

Non-ischemic dilated cardiomyopathy (NIDCM) is characterized by ventricular dilation and reduced myocardial contractile function in the absence of obstructive coronary artery disease [1]. Once diagnosed, these patients are recognized to be at increased risk of heart failure and sudden cardiac death (SCD) [2]. In the majority of cases, the underlying etiology of NIDCM remains unknown and therefore management is focused towards optimization of medical therapy and the appropriate use of device therapy, inclusive of implantable cardioverter-defibrillators (ICD) and cardiac resynchronization therapy (CRT) [3]. To date, therapeutic decision making for ICD therapy remains solely reliant on the estimation of left ventricular ejection fraction (LVEF) and, accordingly, serves as the primary metric of prognostication in NIDCM patients [3].

Recently, the presence and pattern of myocardial fibrosis, as assessed by late gadolinium enhancement (LGE) cardiovascular magnetic resonance (CMR), has been shown to provide incremental prognostic value for the prediction of major adverse cardiovascular outcomes in patients with NIDCM [4]. Such studies have focused on expert subjective scoring of a mid-wall “striae” pattern of LGE, emerging as the most prognostic pattern of LGE in this population [4–7]. While the pathophysiology of this phenomenon remains uncertain, the presence of signal enhancement in the septal segments using this technique has been histologically associated with replacement fibrosis and clinically associated with elevated rates of heart failure hospitalization, heart transplantation, and SCD [4, 7]. Despite strong clinical value, the translation of this marker into clinical practice at non-expert imaging sites remains limited, as objective diagnostic criteria have not been established. Emphasizing such need, recent reports have suggested that subtle elevations in signal in the most basal septum may be physiologic (i.e. local vascular structures, annular fibrosis, etc.) and unrelated to objective markers of cardiovascular disease [8]. Therefore, the development and validation of objective, quantitative criteria for pathologic septal fibrosis is of critical importance to the field.

In this study we evaluated the capacity of novice (non-MD) readers to use a standardized approach to the quantification of septal fibrosis in patients with NIDCM and describe the predictive performance of threshold-based definitions versus expert visual interpretation for the prediction of cardiac death and appropriate ICD therapy.

## Methods

### Study population

NIDCM patients (≥18 years of age) consecutively enrolled in a prospective clinical outcomes registry of clinically referred patients undergoing CMR were identified. NIDCM was defined as the absence of obstructive coronary artery disease (≥70 % stenosis of ≥1 epicardial coronary vessel, or ≥50 % of the left main coronary artery) by cardiac catheterization, no prior history of myocardial infarction, and no subendocardial (ischemic) pattern of LGE. Patients with a diagnosis of hypertrophic cardiomyopathy, sarcoidosis, amyloidosis, or arrhythmogenic right ventricular cardiomyopathy were excluded. No patient with a glomerular filtration rate <30 ml/min/1.76 m^2^ was included.

This study was approved by the local institutional ethics review board and all subjects provided written informed consent.

### CMR Protocol

CMR studies were performed using a 3-T scanner (Siemens, Erlangen, Germany) and 32-element coil. The imaging protocol included standard cine and LGE imaging, the latter performed using a standard inversion recovery gradient echo pulse sequence 10 min following intravenous gadolinium contrast (0.15–0.2 mmol/kg; Gadovist; Bayer, Inc). Typical imaging parameters were slice thickness 8 mm, gap 2 mm, TE 1.93 ms, flip angle 20°, matrix 256 × 205. Images were acquired at end-expiration with the use of ECG gating.

### CMR image analysis

Left ventricular volumes and mass were calculated by blinded core laboratory personnel using commercially available software (cvi^42^; Circle Cardiovascular Inc., Calgary, Canada). This was performed on sequential short axis cine images with semi-automated tracing of the endocardial and epicardial contours, deriving LV end-diastolic volume (LVEDV), LV end-systolic volume (LVESV), ejection fraction (EF) and LV mass, as previously described [7]. LV volumes and mass were indexed to body surface area.

LGE imaging was interpreted using two separate approaches; expert visual scoring and novice quantitative scoring. The former, performed by an experienced CMR expert (JAW), characterized the presence or absence of non-ischemic LGE and its geographic distribution(s), as follows; septal (mid-wall striae), RV insertion site (RVI), sub-epicardial, and diffuse. In contrast, the latter was performed by undergraduate students with no prior experience in CMR interpretation, trained to use commercial signal threshold-based LGE analysis software (cvi^42^; Circle Cardiovascular Inc., Calgary, Canada).

**Fig. 1:**
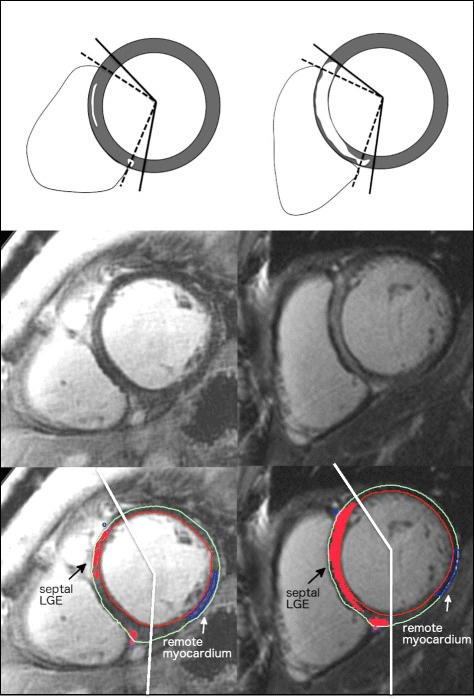
Examples of quantitative septal LGE analysis. *Left*: Patient with visually scored septal LGE but low quantitative LGE burden. No events were recorded. *Right*: Patient with visually scored septal LGE and high quantitative LGE burden. An appropriate ICD therapy was subsequently delivered for fast VT. LGE = late gadolinium enhancement; ICD = implantable cardioverter-defibrillator; VT = ventricular tachycardia

Signal threshold-based LGE analysis was performed using sequential short axis LGE images to obtain both total (ie: whole heart) LGE, as previously described [9], and septal segment LGE burden. The latter employed a standard exclusion tool (typically used to exclude artifact) to focus LGE quantification to the septal segments, as shown in Fig. 1. The septal region was *a priori* defined as the myocardium between, and inclusive of the RV insertion regions. Borders of the RV insertion regions were set at 10 mm beyond the point of contact between the RV and LV walls (Fig. 1). A Signal Threshold versus Reference Mean (STRM) approach was chosen with three thresholds tested, these being >5SD, 3SD, >2SD above the mean of remote reference myocardium. The Full Width at Half Maximum (FWHM) approach was purposely avoided as novice interpreters are not expected to identify the septal fibrosis required to reference this technique. Total LGE and septal LGE burden were expressed as absolute mass (g) and percentage of the LV mass (%).

**Fig. 2:**
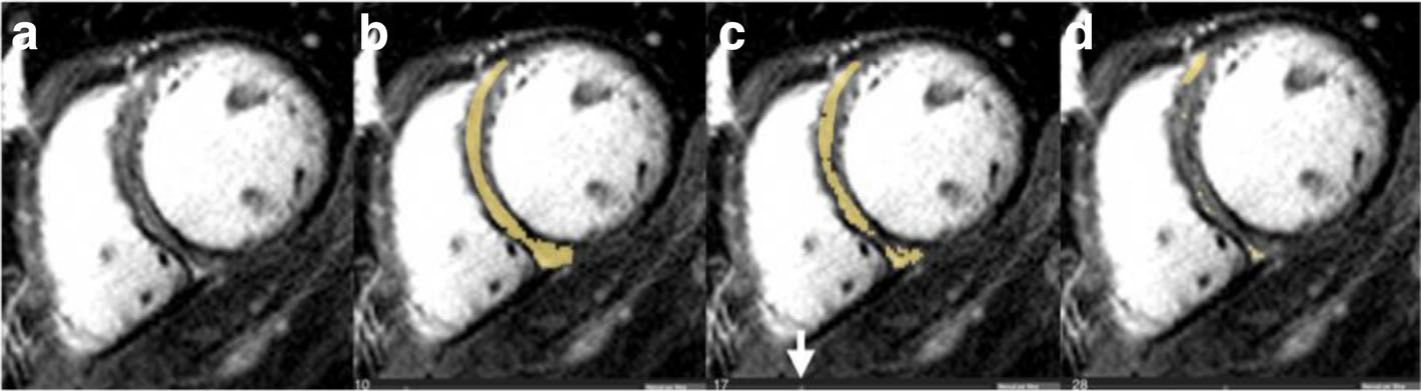
LGE quantification by expert using manual adjustment of the SI threshold. Absolute SI threshold being adjusted (*arrow*) with tissue having a signal above the selected value highlighted in *yellow*. Panel **a**: raw LGE image. Panel **b**: At 10 units this was felt to over-represent LGE. Panel **c**: At 17 units an appropriate segmentation was achieved. Panel **d**: At 28 units an under-representation of LGE was seen. Therefore, 17 units was chosen to represent LGE burden. LGE = late gadolinium enhancement; SI = signal intensity

To provide objective comparison of expert visual interpretation of septal fibrosis versus STRM threshold-based techniques, we performed a separate analysis inclusive of expert manual threshold LGE quantification in 30 randomly selected patients of the same cohort. Following the application of septal segment borders, an expert (YM) manually adjusted the signal intensity threshold for each LGE slice until visual agreement with fibrosis distribution was achieved as previously described (Fig. 2) [10]. Total septal fibrosis estimates obtained using expert-defined signal thresholding were compared to those obtained by the STRM >5SD, 3SD and 2SD techniques for each patient.

Intra- and inter-observer reproducibility was assessed for STRM-based quantitative LGE analysis. Two blinded observers independently analyzed LGE images from 15 randomly selected patients on two separate occasions.

### Clinical follow-up and cardiovascular events

All patients were followed from the time of CMR using scripted telephone interviews, review of medical records, and a blinded review of ICD device interrogations. The primary composite endpoint was cardiac death or appropriate ICD therapy. Appropriate ICD therapy was defined as anti-tachycardia pacing (ATP) and/or shock for confirmed sustained fast ventricular tachycardia (R-R <320 ms) or ventricular fibrillation, as adjudicated by a blinded electrophysiologist. ICD therapy was deemed inappropriate when provided in the setting of supra-ventricular tachycardia, T-wave over-sensing or for device malfunction (such as wire fracture). Cardiac death was defined as death attributable to underlying cardiomyopathy and included SCD or death due to pump failure. The secondary composite endpoint was appropriate ICD therapy or SCD and was selected to provide an arrhythmia focused clinical endpoint. SCD was defined as death occurring within 1 hour of symptom onset. Complete follow-up was available for all patients.

### Statistical analysis

Continuous data was expressed as means with standard deviation. Categorical variables were expressed in counts with percentages. Comparison between groups was performed with the 2-sample Student *t*-test for continuous, normally distributed data and Wilcoxon rank sum test for continuous, non-normally distributed data. Fisher’s exact test was used to compare categorical data. Univariable associations with the primary and secondary outcomes were performed using Cox proportional hazards regression, and event-free survival, stratified by extent of LGE, was estimated with Kaplan-Meier survival methods. Evaluation of independent associations between qualitative and quantitative septal LGE for the primary composite endpoint was addressed by addition to a multivariable model containing established risk predictors of LVEF *a priori*. All models were assessed for collinearity and proportional hazards assumption. Net reclassification index was performed to evaluate the incremental value of quantitative septal LGE to the baseline multivariable model (age, LVEF). Receiver-operator characteristic (ROC) curves were constructed to determine the optimal cutoff value for quantitative septal LGE assessment to predict the primary endpoint. Comparisons between visually guided (expert) and STRM-based signal thresholds for LGE segmentation were performed using Bland-Altman analysis and intra-class correlation coefficients (ICC). All statistical analysis was performed using SPSS for Macintosh, version 21.0 (SPSS Inc., Chicago, Illinois), Stata/SE 12.0 (StataCorp LP, College Station, Texas) and SAS (SAS Institutes, version 9.4, Cary, North Carolina).

## Results

### Baseline clinical characteristics

A total of 118 patients with NIDCM were studied. Baseline clinical characteristics are shown in Table 1. The mean age of the population was 57 ± 14 years with 42 % being female. A total of 56 patients (47 %) received ICD device implantation. Patients experiencing a primary outcome were more likely to have device implantation (85 % versus 40 %, *p* < 0.001) and greater use of diuretics (100 % versus. 63 %, *p* < 0.01), otherwise being similar with respect to non-CMR based characteristics.

**Table 1:** Baseline demographics of the cohort and stratified by primary outcome measure

Characteristics	Total cohort (*N* = 118)	With primary outcome (*N* = 20)	Without primary outcome (*N* = 98)	*P*-value
Age in years	57 ± 14	61 ± 13	57 ± 14	0.23
Female, n (%)	50 (42)	9 (45)	41 (42)	0.81
BMI	29 ± 6	30 ± 7	29 ± 6	0.90
Hypertension, n (%)	48 (41)	11 (55)	37 (38)	0.21
Diabetes, n (%)	20 (17)	6 (30)	14 (14)	0.11
Hyperlipidemia, n (%)	40 (34)	8 (40)	32 (33)	0.61
Smoking, n (%)	39 (33)	6 (30)	33 (34)	1.0
QRS interval (ms)	138 ± 31	144 ± 26	137 ± 32	0.32
QTc (ms)	469 ± 42	472 ± 37	469 ± 44	0.54
Device implantation, n (%)	56 (47)	17 (85)	39 (40)	<0.001*
NYHA functional class, n(%)				0.05
I	12 (10)	0(0)	12(12)	
II	51 (43)	6 (30)	45 (46)	
III	46 (39)	13 (65)	33 (34)	
IV	9 (8)	1 (5)	8 (8)	
Medications^a^				
ACE inhibitor or ARB	86 (85)	17 (94)	69 (83)	0.30
Amiodarone	5 (5)	1 (6)	4 (5)	1.0
Beta-blocker	89 (88)	17 (94)	72 (87)	0.69
Calcium Blocker	7 (7)	2 (11)	5(6)	0.61
Digoxin	22 (22)	5 (28)	17 (20)	0.53
Diuretics	70 (69)	18 (100)	52 (63)	<0.01*
Statin	40 (40)	6 (33)	34 (41)	0.61

Continuous data are expressed as mean ± SD, categorical data as n (%). *p < 0.05

*Abbreviations*: *BMI* Body mass index, *NYHA* New York Heart Association, *ACE* Angiotensin converting enzyme, *ARB* Angiotensin II receptor blocker. ^a^Total number of the patients for the medications data is 101

### Primary and secondary outcomes

During a median follow-up period of 1.9 years (IQR = 2.0) (mean 2.1 ± 1.3 years), a total of 20 patients (17 %) experienced the primary outcome. Of these, seven patients suffered cardiac death while 14 patients received an appropriate ICD device therapy. One patient experienced both appropriate ICD therapy and cardiac death (the former used for statistical analysis). A total of 15 (13 %) patients experienced the secondary arrhythmic endpoint (1 SCD and 14 appropriate ICD device therapies). An additional five (4.2 %) patients died from non-cardiac causes.

### CMR characteristics

CMR-based findings are shown in Table 2. Volumetric quantification showed a mean LVEF of 32 ± 12 % with a mean LVEDVI of 119 ± 42 ml/m^2^. Expert interpretation identified any LGE in 66 (56 %) patients with the prevalence of each LGE pattern as follows; 37 (31 %) had septal striae fibrosis, 38 (32 %) had septal RV insertion site fibrosis, and 19 (16 %) had a sub-epicardial pattern of fibrosis. Objective LGE quantification found mean total LGE mass and percent LGE (of LV mass) by each STRM threshold as follows; >5SD: 5.5 ± 10.4 g and 5.6 ± 9.0 %; >3SD: 13.6 ± 16.5 g and 14.3 ± 14.2 %; >2SD: 23.0 ± 20.3 g and 24.5 ± 16.8 %, respectively. The mean septal LGE mass and percent (of LV mass) by each STRM threshold were; 5SD: 2.7 ± 3.4 g, 2.9 ± 3.6 %; 3SD: 6.5 ± 6.1 g, 6.9 ± 6.3 %; 2SD: 10.4 ± 7.7 g, 11.1 ± 7.5 %, respectively.

**Table 2:** CMR Characteristics of the Cohort and Stratified by Primary Outcome Measure

Characteristics	Total cohort (*N* = 118)	With primary outcome (*N* = 20)	Without primary outcome (*N* = 98)	*P*-value
LVEDVI (ml/m^2^)	119 ± 42	128 ± 32	117 ± 43	0.12
LVESVI (ml/m^2^)	84 ± 40	97 ± 33	82 ± 41	0.06
LVEF (%)	32 ± 12	26 ± 11	33 ± 12	0.02*
LV mass index (g/m^2^)	95 ± 29	92 ± 28	96 ± 30	0.58
RVEDVI (ml/m^2^)	69 ± 23	77 ± 20	68 ± 24	0.09
RVESVI (ml/m^2^)	40 ± 22	47 ± 19	38 ± 23	0.02*
RVEF (%)	45 ± 17	40 ± 16	46 ± 17	0.10
LGE – Qualitative (Visual)				
Any LGE, n (%)	66 (56)	16 (80)	50 (51)	0.03*
Septal LGE, n (%)	37 (31)	11 (55)	26 (27)	0.02*
RV insertion site LGE, n (%)	38 (32)	10 (50)	28 (29)	0.07
Septal and/or RV insertion site LGE, n (%)	55 (47)	15 (75)	40 (41)	<0.01*
LGE – Quantitative				
Total LGE -5SD (%)	5.6 ± 9.0	7.4 ± 9.4	5.2 ± 8.9	0.22
Total LGE -3SD (%)	14.3 ± 14.2	18.8 ± 15.1	13.3 ± 13.9	0.11
Total LGE -2SD (%)	24.5 ± 16.8	31.1 ± 17.8	23.1 ± 16.4	0.05
Septal LGE -5SD (%)	2.9 ± 3.6	5.4 ± 5.0	2.4 ± 3.0	<0.01*
Septal LGE -3SD (%)	6.9 ± 6.3	11.7 ± 8.5	5.9 ± 5.2	<0.01*
Septal LGE -2SD (%)	11.1 ± 7.5	16.9 ± 9.5	9.9 ± 6.5	<0.01*

Continuous data are expressed as mean ± SD, categorical data as n (%). *p < 0.05

*Abbreviations*: *LV* Left ventricular, *EDVI* End diastolic volume indexed to body surface area, *ESVI* End systolic volume indexed to body surface area, *EF* Ejection fraction, *RV* Right ventricular, *LGE* Late gadolinium enhancement

LGE was quantified by signal threshold versus reference mean (STRM) technique using signal intensity threshold of 5SD, 3SD and 2SD of the remote myocardium. Total LGE and septal LGE burden were expressed as percentage of the LV mass (%)

CMR-based characteristics for patients with and without primary outcomes are similarly shown in Table 2. Patients experiencing a primary outcome had reduced LVEF (26 ± 11 versus 33 ± 12 %, *p* < 0.05) and increased RVESVI (47 ± 19 versus 38 ± 23 ml, *p* = 0.02) versus those without a primary outcome. LGE-based variables showed patients experiencing the primary outcome to be more likely to have any LGE (80 % versus 51 %, *p* = 0.03), a septal striae pattern of LGE (55 % versus 27 %, *p* = 0.02) and a septal striae pattern and/or RV insertion site LGE (75 % versus 41 %, *p* < 0.01).

Quantitative LGE analysis by STRM 2SD method revealed a strong trend for total LGE to be elevated among patients experiencing the primary outcome (31.1 ± 17.8 % versus 23.1 ± 16.4 %, *p* = 0.05). By comparison, septal LGE burden by all the STRM methods was significantly elevated among patients experiencing the primary outcome (5SD 5.4 ± 5.0 % versus 2.4 ± 3.0 %, *p* < 0.01, 3SD 11.7 ± 8.5 % versus 5.9 ± 5.2 %, *p* < 0.01, 2SD 16.9 ± 9.5 % versus 9.9 ± 6.5 %, *p* < 0.01)

**Fig. 3:**
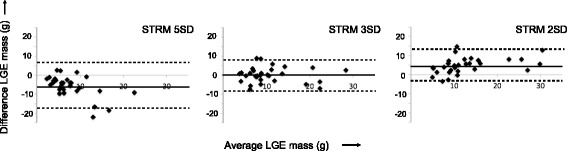
Bland-Altman plots between each semi-automated signal threshold versus reference mean (STRM) technique and expert, visually guided thresholding of septal LGE. The STRM >3SD technique showed greatest agreement with expert opinion (mean difference and 95 % limits of agreement = 0.2 ± 8.0 g). LGE = late gadolinium enhancement

Of all signal-threshold techniques, STRM >3SD was found to best approximate expert visual interpretation (using a manually-adjusted signal threshold). This is illustrated by Bland-Altman analyses performed for septal LGE volumes obtained by each semi-automated technique versus expert visual thresholding (Fig. 3). The STRM > 3SD technique achieved an ICC of 0.8 with mean difference of 0.2 ± 8.0 g.

### Univariable predictors of the primary outcome

No baseline clinical characteristic reached statistical significance in the univariable associations with the primary composite outcome. Univariable and multivariable associations of CMR-based characteristics with the primary outcome are shown in Table 3. Of the non-LGE based characteristics, both LVEF and LVESVI were significantly associated with the primary outcome. Of LGE based characteristics, expert-based visual identification of any LGE, mid-wall septal striae LGE, RV insertion (RVI) site LGE, and septal and/or RVI site LGE were each associated with the primary outcome. Of these, the visual presence of septal and/or RVI site fibrosis was the strongest predictor of the primary outcome (HR 5.34, *p* = 0.001). By comparison, all novice-based quantitative measures for total and septal LGE volume were significantly associated with the primary outcome, the only exception being total LGE by the STRM >5SD technique. Among all quantitative measures, septal LGE obtained using the STRM >5SD technique was the strongest predictor of the primary outcome (HR 1.21 per 1 %, *p* < 0.001).

**Table 3:** Univariable and multivariable associations of CMR characteristics with primary composite outcome

Characteristics	Univariable				Model 1^a^				Model 2^b^				Model 3^c^			
	HR	95 % CI	*χ* ^2^	*P* value	HR	95 % CI	*χ* ^2^	*P* value	HR	95 % CI	*χ* ^2^	*P* value	HR	95 % CI	*χ* ^2^	*P* value
CMR – non LGE variables																
LVEDVI (per 1 ml/m^2^)	1.01	1.00–1.02	3.77	0.05												
LVESVI (per 1 ml/m^2^)	1.01	1.00–1.02	6.14	0.02*												
LVEF (per 1 %)	0.93	0.89–0.98	9.86	0.003*	NA	NA	NA	NS	0.95	0.91–0.996	25.40	0.04*	NA	NA	NA	NS
LV mass index (per 1 g/m^2^)	1.00	0.99–1.02	0.03	0.87												
CMR – LGE variables																
Visual – Any LGE	4.49	1.48–13.65	8.33	0.008*												
Visual – septal LGE	4.41	1.75–11.11	11.72	0.002*												
Visual – RVI LGE	2.61	1.08–6.32	4.90	0.033*												
Visual – septal or RVI LGE	5.34	1.91–14.93	12.53	0.001*	5.34	1.91–14.93	12.53	0.001*								
Quantitative-total LGE (per 1 %) 5SD	1.03	0.99–1.06	2.03	0.17												
Quantitative-total LGE (per 1 %) 3SD	1.02	1.00–1.04	4.42	0.04*												
Quantitative-total LGE (per 1 %) 2SD	1.02	1.00–1.05	5.65	0.02*												
Quantitative-septal LGE (per 1 %) 5SD	1.21	1.1–1.31	23.60	<0.001*					1.17	1.07–1.28	25.40	0.001*				
Quantitative-septal LGE (per 1 %) 3SD	1.12	1.07–1.18	22.22	<0.001*												
Quantitative-septal LGE (per 1 %) 2SD	1.11	1.06–1.17	20.47	<0.001*												
% Septal LGE 5SD >2.74 %	8.65	3.06–24.51	23.12	<0.001*									8.65	3.06–24.51	23.12	<0.001*
% Septal LGE 3SD >6.63 %	5.68	2.11–15.28	14.81	0.001*												
% Septal LGE 2SD >10.15 %	6.09	2.12–17.50	14.24	0.001*												

*Abbreviations*: *LV* Left ventricular, *EDVI* End diastolic volume indexed to body surface area, *ESVI* End systolic volume indexed to body surface area, *EF* Ejection fraction, *LGE* Late Gadolinium Enhancement, *RVI* Right ventricular insertion site. LGE was quantified by signal threshold versus reference mean (STRM) technique using signal intensity threshold of 5SD, 3SD and 2SD of the remote myocardium. *p < 0.05

^a^Model 1 represents multivariable Cox regression model with LVEF, and qualitative septal or RVI LGE (present or absent)

^b^Model 2 represents multivariable Cox regression model with LVEF, and quantitative septal LGE 5SD

^c^Model 3 represents multivariable Cox regression model with LVEF, and quantitative septal LGE 5SD cut off >2.74 %

### Multivariable predictors of the primary outcome

Multivariable Cox regression analysis was performed to identify independent associations with the primary composite outcome using *a priori* parameters of LVEF (Table 3), followed by the separate addition of expert-based visual septal and/or RVI LGE (Model 1; Table 3) and novice-based septal LGE (STRM >5SD) volume (Model 2; Table 3). Both models demonstrated independent associations with the primary composite outcome with corresponding adjusted HR of 5.34 (95 % CI 1.91–14.93, *p* = 0.001) for expert visual interpretation and 1.17 per percent (95 % CI 1.07–1.28, *p* = 0.001) for novice-based septal LGE volume.

ROC analyses were performed to identify optimal quantitative thresholds for septal LGE volume (% LV mass) based on combinations of sensitivity and specificity to predict the primary composite outcome. The following thresholds were identified; 2.74 % for STRM 5SD, 6.63 % for STRM 3SD and 10.15 % for STRM 2SD.

Using those optimal thresholds, the sensitivity, specificity and numbers of patients missed (i.e. negative for the test but had an event) were as follows: 5SD: 70, 74 %, and six patients; 3SD: 70, 69 %, and six patients; 2SD: 75, 64 %, and five patients. The positive predictive value (PPV) and negative predictive value (NPV) for each threshold were; 5SD: 36, 92 %; 3SD: 32, 92 %; 2SD: 30, 93 %, respectively.

Univariable analysis revealed the STRM >5SD technique to provide the strongest HR for prediction of the primary outcome (HR 8.65, *p* < 0.001). Accordingly, a final multivariable Cox regression analysis was performed inclusive of LVEF, and >2.74 % septal LGE by STRM >5SD (Model 3, Table 3). In this model only the latter marker was an independent predictor of the primary outcome, providing a 9-fold higher risk of cardiac death or appropriate ICD therapy (95 % CI 3.06–24.51, *p* < 0.001).

**Fig. 4:**
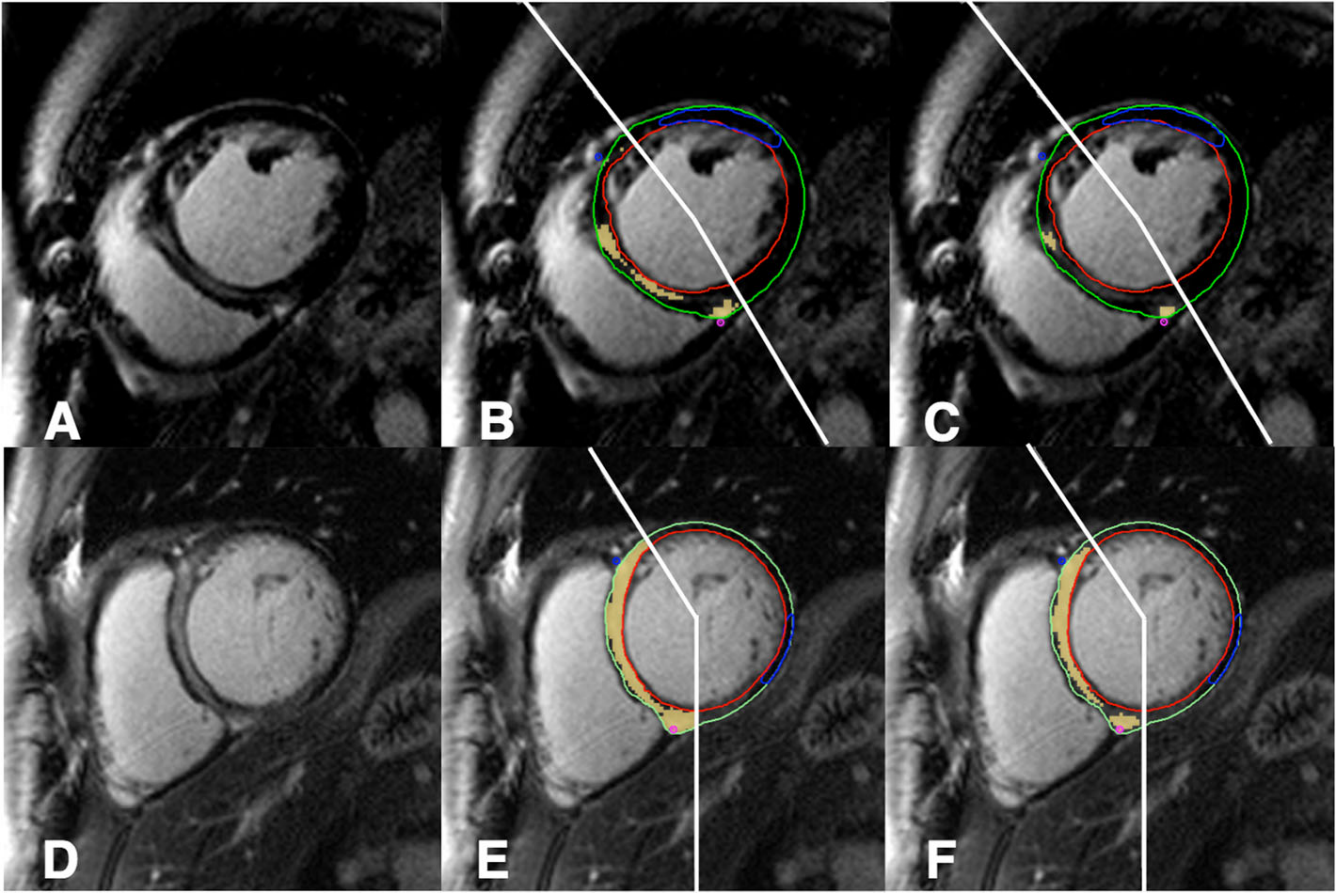
Top row; A case with expert visual scoring of septal LGE (**a**), high quantitative LGE burden using STRM >3SD (10.0 % of LVmass, **b**) but low LGE burden by STRM >5SD (2.1 % of LV mass, **c**). No events were recorded. Bottom row; A case with expert visual scoring of septal LGE (**d**) and high quantitative LGE burden by both STRM >3SD (35.6 % of LV mass, **e**) and STRM >5SD (21.5 % of LV mass, **f**). An appropriate ICD therapy was subsequently delivered for fast VT. LGE = late gadolinium enhancement; STRM = signal threshold versus reference mean; ICD = implantable cardioverter-defibrillator

Relevant patient examples are provided in Fig. 4.

### Univariable predictors of the secondary outcomes

LVEF, LVEDVI, LVESVI and RVEDVI were significantly associated with the secondary outcome (HR 0.94, 95 % CI 0.89–0.99, *p* = 0.01; HR 1.01, 95 % CI 1.00–1.03, *p* = 0.02; HR = 1.02, 95 % CI 1.00–1.03, *p* = 0.01, HR 1.02, 95 % CI 1.00–1.05, *p* = 0.02, respectively). Expert visual scoring of any LGE, septal striae LGE, RVI LGE, and septal and/or RVI LGE were each significantly associated with the secondary outcome (HR 4.76, 95 % CI 1.32–17.22, *p* = 0.02; HR 6.08, 95 % CI 2.0–18.48, *p* = 0.001; HR 3.10, 95 % CI 1.12–8.63, *p* = 0.03; HR 7.56, 95 % CI 2.09–27.37, *p* = 0.002, respectively). Using the STRM >5SD technique, % total LGE was not significantly associated with the secondary outcome, however, % septal LGE was significantly associated (HR 1.22, 95 % CI 1.11–1.34, *p* < 0.001).

### Annual event rates and survival analysis

**Fig. 5:**
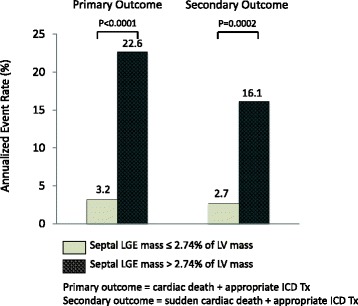
Annual event rates for primary and secondary composite outcomes in patients with quantitative septal LGE above and below a 2.74 % threshold. Patients with septal LGE greater than 2.74 % had a significantly higher annual event rate than those without for the primary outcome (22.6 % versus 3.2 %, *p* < 0.0001), and secondary outcome (16.1 % versus 2.7 %, *p* < 0.0002). LGE = late gadolinium enhancement

**Fig. 6:**
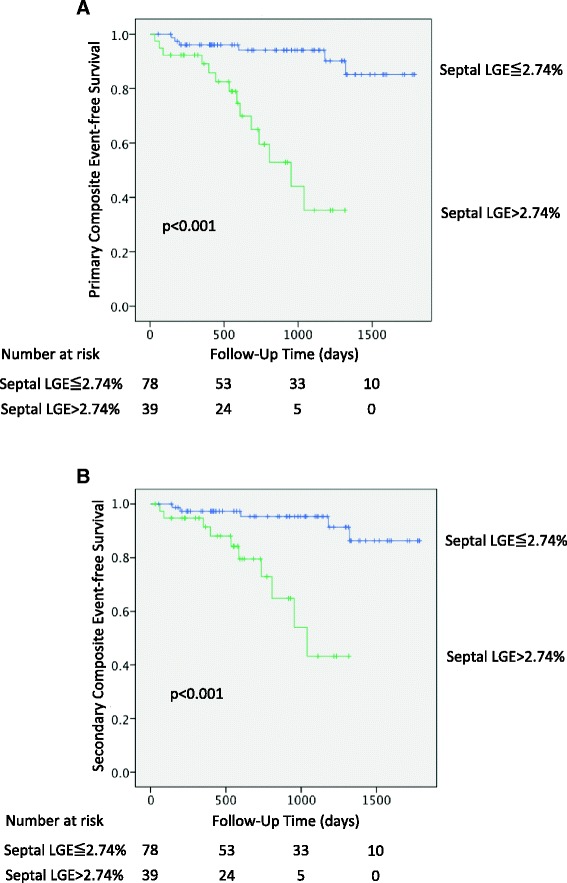
Kaplan-Meier survival analysis for the primary and secondary outcome stratified by quantitative septal LGE above and below 2.74 %. Patients with septal LGE greater than 2.74 % had lower event-free survival for the primary outcome of cardiac death or appropriate ICD therapy (*p* < 0.001, **a**). Similar findings for the secondary endpoint of sudden cardiac death or appropriate ICD therapy were found (*p* < 0.001, **b**). LGE = late gadolinium enhancement

Annual event rates for the primary and secondary arrhythmic outcomes, stratified according to the STRM >5SD septal LGE threshold are shown in Fig. 5. Patients meeting these objective criteria had significantly higher annual event rates for both the primary (22.6 % versus 3.2 %, *p* < 0.0001) and secondary (16.1 % versus 2.7 %, *p* < 0.0002) composite outcome measures. Kaplan-Meier survival curves stratified by the same septal LGE threshold are shown in Fig. 6 for both the primary (Fig. 6a) and secondary arrhythmic (Fig. 6b) composite outcome measures. Significantly lower event-free survival was identified for both the primary and secondary outcomes (*p* < 0.001 for both).

**Fig. 7:**
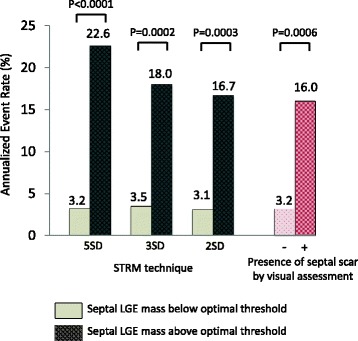
Annual event rates for primary endpoint in patients with quantitative septal LGE above and below the optimal threshold for each STRM technique (5SD 2.74 %, 3SD 6.63 %, 2SD 10.15 % of LV mass) and patients with and without septal scar by visual assessment. The patients with septal LGE greater than the optimal threshold had a significantly higher annual event rate than those without for all the STRM technique, and it was the most prominent for STRM 5SD technique (3.2 % vs 22.6 %, *p* < 0.0001). LGE = late gadolinium enhancement, STRM = signal threshold versus reference mean

The relative performance of threshold-based quantification versus expert visual scoring for prediction of the primary outcome is shown in Fig. 7. Using optimal thresholds, the patients with septal LGE greater than the threshold had a significantly higher annual event rate than those without for all STRM-based techniques, and was most prominent for the STRM >5SD technique (22.6 % vs 3.2 %, *p* < 0.0001). Corresponding event rates in patients with versus without septal fibrosis by expert visual scoring was 16.0 % vs 3.2 % (*p* = 0.0006).

### Improvement of risk discrimination

The addition of quantitative septal LGE (STRM >5SD), using a cutoff of >2.74 %, to baseline risk modeling inclusive of LVEF significantly improved risk reclassification (net reclassification index 0.54, 95 % CI 0.16–0.92, *p* = 0.005).

### Intra- and inter-observer reproducibility

**Fig. 8:**
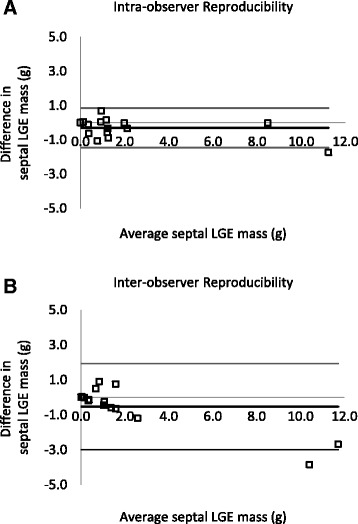
Intra- and inter-observer reproducibility testing results described using Bland-Altman analysis. Intra-observer (**a**) and inter-observer (**b**) analysis produced an ICC of 0.983 (95 % CI, 0.951–0.994) and 0.943 (95 % CI, 0.838–0.980), respectively

Intra and Inter-observer reproducibility analyses showed strong agreement for septal LGE quantification. The STRM >5SD technique produced an ICC of 0.983 (95 % CI, 0.951–0.994) and 0.943 (95 % CI, 0.838–0.980), respectively. Corresponding Bland-Altman analyses are shown in Fig. 8.

## Discussion

This study demonstrates clinical feasibility, high reproducibility, and powerful prognostic utility of objective criteria for septal LGE in the prediction of major cardiovascular events in NIDCM. Using an objective threshold, novice readers were able to identify patients with a 9-fold higher risk of cardiac death or appropriate ICD therapy versus expert readers who, using conventional visual interpretation, achieved 5-fold risk discrimination. This objective marker provided robust risk reclassification (NRI 0.54, 95 % CI 0.16–0.92, *p* = 0.005) and also meaningfully discriminated patients at very high versus low risk of the primary (22.6 % versus 3.2 %, *p* < 0.0001) and secondary (16.1 % versus 2.7 %, *p* < 0.0002) composite outcomes.

Autopsy based studies have identified replacement myocardial fibrosis, interstitial fibrosis and cellular hypertrophy in patients with NIDCM [11, 12]. Of these, conventional LGE CMR imaging allows for the non-invasive detection and quantification of replacement fibrosis [4], a phenomenon now recognized to predominantly occur in the septal myocardial segments, and thought to represent adverse remodeling related to increased wall stress and biomechanical strain [13]. Several studies have supported associations between expert visual scoring of LGE and future cardiovascular events in this population [4, 7, 14–18]. Specifically, four key studies identified a mid-wall septal mid-wall “striae” pattern of LGE to be the strongest predictor of future events [4, 7, 14, 18]. In all of these studies, expert readers (at luminary CMR sites) performed subjective visual scoring of septal LGE making clinical dissemination of this marker challenging. While objective assessments of global LGE was performed in two of these studies [16, 17], patients with NIDCM frequently demonstrate multiple geographic patterns of LGE that may not contribute significantly to the development of cardiovascular events [7]. Therefore, objective and reproducible criteria for the classification of septal LGE, particularly one appropriate for use by non-expert readers, is highly desirable.

Of studies to date, consistent findings have been reported for the prevalence of septal LGE in cohorts with NIDCM and its prediction of future cardiovascular events. As a sentinel study, Assomull, et al.*,* described 101 patients with NIDCM and followed them over a median of 658 days for the composite primary endpoint of all-cause mortality or cardiac-related hospitalization [14]. The visual interpretation of a mid-wall striae pattern of LGE was associated with a HR of 3.4 (*p* = 0.01) for the primary outcome and was the sole predictor following multivariable analysis (HR 3.1, *p* = 0.03). In this study global but not septal LGE burden was assessed using a threshold of >2SD STRM technique. While the global measure was associated with the primary outcome (HR 1.12 per 1 %, *p* = 0.02), an ROC based threshold did not achieve statistical significance for the primary endpoint (*p* = 0.07). Gulati, et al. studied 472 patients with NIDCM and similarly identified that expert visual reporting of a mid-wall striae LGE pattern was the strongest independent predictor of all-cause mortality (HR 2.4, *p* < 0.001) following adjustment for LVEF and other prognostic variables [4]. Total LGE was quantified using a FWHM technique, one that relies on expert opinion to identify and reference fibrosis. Accordingly, it is not unexpected that this measure was similarly associated to the composite primary outcome (HR 1.11 per 1 %, *p* < 0.001). An objective threshold value for the prediction of events was not tested.

While not focused on septal LGE quantification, three additional studies have evaluated global LGE quantification in patients with NIDCM [15–17]. Wu, et al. studied 65 patients with NIDCM undergoing primary prevention ICD. In this high-risk cohort, total LGE, measured using an STRM >2SD technique, showed a difference in risk between those above the median value (HR 11.9) versus below the median value (HR 6.7) for the primary composite outcome of cardiac death, appropriate ICD therapy and heart failure requiring hospitalization [15]. However, the elevated HR of below the median threshold led to conclusions that global measures of LGE may not adequately differentiate a low risk cohort from those at elevated risk of events. Peazzolo-Marra, et al. studied 137 patients for the composite arrhythmic outcome of sustained VT, VF, appropriate ICD therapy, or SCD and demonstrated total LGE extent, performed using the same technique, did not show predictive utility (*p* = 0.18) [17]. Finally, Neilan, et al. studied 162 patients following them for the composite endpoint of cardiac death or appropriate ICD therapy [16]. In this study total LGE extent was found to be predictive of the primary outcome when estimated using either the STRM >2SD (HR 1.15 per 1 %, *p* < 0.0001) or FWHM (HR 1.16 per 1 %, *p* < 0.0001) technique. Overall, studies to date have provided inconsistent findings regarding the prognostic value of global LGE in NIDCM.

The current study focused on validating a standardized approach to the definition of septal LGE in patients with NIDCM, a finding that has been consistently associated with future cardiovascular events when scored by expert readers from luminary CMR centers. Using the described approach, the presence of significant septal LGE (approximately 3 % or more of the LV mass) identified patients with a 9-fold higher risk of cardiac death or appropriate ICD therapy. This performance was achieved by non-expert readers with no prior experience in viewing CMR images, and exceeded the predictive utility of expert visual interpretation. Further, this predictive utility was maintained for the arrhythmia-focused composite secondary outcome, one relevant to clinical decision-making surrounding ICD candidacy.

It is intriguing that we identified expert visual evaluation of septal LGE to most closely approximate the STRM >3SD technique, inherently being a more sensitive approach to identification of this marker. Consistent with this finding, the predictive utility of expert visual interpretation best matched that of the STRM >3SD technique. The fact that a higher threshold technique (STRM >5SD) was found to better predict cardiovascular events raises two important postulates. First, it has been described that non-pathological forms of basal septal LGE exist across the general population and would therefore be inclusive of those ultimately developing NIDCM. These patterns show no association with other measures of cardiovascular disease [8], have a lower signal intensity profile, but may still garner attention from expert interpreters. Second, while a spectrum of signal is expected to occur with increasing pathological forms of septal fibrosis, greater density of fibrosis (ie: exceeding the 5SD threshold) may be more likely to provoke cardiovascular events. This might be particularly expected for arrhythmic events where dense replacement fibrosis is well established to provoke re-entry mechanism ventricular arrhythmias.

## Limitations

Several limitations to our study must be recognized, including its single center design and limited sample size. First, appropriate ICD therapy can be considered an imperfect clinical outcome given that documented therapies may not be equivalent to life threatening arrhythmic events. Indeed, large-scale clinical trials have shown higher rates of appropriate ICD therapy in comparison to SCD [19]. To minimize such a bias, we used a primary composite endpoint that included the more robust clinical outcome of cardiac mortality. Second, our study did not evaluate novel emerging CMR techniques, such as T1 mapping, for the evaluation of extracellular volume fraction assessment [20]. These may provide incremental value to risk stratification in this population when added to quantitative LGE assessment of replacement fibrosis, however require ongoing refinement. Finally, while our study considered common baseline clinical and CMR characteristics available in routine practice, many additional factors may uniquely converge for individual patients to contribute to their arrhythmia risk. Accordingly, future studies examining the capacity of data modeling and algorithmic modeling (i.e. machine learning) to consider broader aspects of patient phenotype are required. To accomplish this, large-scale, multi-center initiatives aimed towards standardized patient phenotyping are necessary to derive and validate such models.

## Conclusions

Using the described methodology, the presence of clinically relevant septal fibrosis can be reproducibly defined by non-expert readers and provides superior risk stratification to expert visual interpretation in patients with NIDCM. The described objective criteria is appropriate for expansive clinical adoption beyond expert sites and should be considered as a validated marker of adverse outcomes for future clinical studies in this population.

## Abbreviations

CMR: Cardiovascular magnetic resonance
EDV: End diastolic volume
EF: Ejection fraction
ESV: End systolic volume
FWHM: Full width at half maximum
ICD: Implantable cardioverter-defibrillator
LGE: Late gadolinium enhancement
LV: Left ventricular
NIDCM: Non-ischemic dilated cardiomyopathy
RV: Right ventricular
RVI: Right ventricular insertion site
SCD: Sudden cardiac death
STRM: Signal threshold versus reference mean
VT: Ventricular tachycardia
